# Seroprevalence of anti-hepatitis E antibodies and antigens among HIV-infected patients in Fars Province, southern Iran

**DOI:** 10.1186/s12985-020-01384-0

**Published:** 2020-07-17

**Authors:** Reza Shahriarirad, Amirhossein Erfani, Mohammad Rastegarian, Ali Zeighami, Nasir Arefkhah, Fariba Ghorbani, Jamal Sarvari, Bahador Sarkari

**Affiliations:** 1 Student Research Committee, School of Medicine, Shiraz University of Medical Sciences, Shiraz, Iran; 2 Department of Parasitology and Mycology, School of Medicine, Shiraz University of Medical Sciences, Shiraz, Iran; 3 Department of Microbiology, School of Medicine, Shiraz University of Medical Sciences, Shiraz, Iran; 4 Gastroenterohepatology Research Center, Shiraz University of Medical Sciences, Shiraz, Iran; 5 Basic Sciences in Infectious Diseases Research Center, Shiraz University of Medical Sciences, Shiraz, Iran

**Keywords:** Hepatitis E, HIV, Seroprevalence, Fars Province, Iran

## Abstract

**Objective:**

HIV-infected patients have immunological and clinical features that might affect the pathogenesis, as well as the outcome of the HIV/HEV co-infection. The current study aimed to determine the seroprevalence of anti-HEV antibodies and HEV antigens among HIV-infected patients in Fars Province, Southwest Iran.

**Methods:**

Blood samples (5 mL) were collected from 251 HIV-confirmed patients. Respective data, including patients’ demographic information, were obtained for each patient. The presence of HEV antigens and anti-HEV antibodies (IgG) were assessed by commercial ELISA kits, based on the manufacturers’ instructions.

**Results:**

Out of 251 cases, 158 (62.9%) were male and 91 (36.3%) were female. Patients’ age varied from 14 to 83 (mean: 40 ± 9.7) years. Out of 251 HIV positive cases, 26 (10.4%) were positive for anti-HEV IgG antibodies and 6 (2.4%) were positive for HEV-antigens. Also, 2 (0.8%) of the patients were positive for both anti-HEV IgG antibodies and antigens. Statistical analysis revealed no significant association between sex and seropositivity to either HEV antigen or antibodies. Moreover, no significant association was seen between age and seropositivity to HEV antigen or antibody (*P* = 0.622 and 0.945, respectively).

**Conclusion:**

Our results showed a relatively low prevalence of HEV-antibodies in HIV-infected patients, indicating that HIV positive patients may not be at greater risk of HEV infection than the general population. Moreover, HEV-antigen was detected in a few cases of HIV-infected individuals which indicate an acute or chronic HEV infection in these patients.

## Background

More than 20 million people are infected with the hepatitis E virus (HEV) around the world, among them about 3.3 million cases are symptomatic that causes 44,000 annual death [1]. Considering the scale of the problem, WHO ranks HEV as the leading cause of global acute viral hepatitis. HIV-infection is accompanied with immunological, epidemiological, and clinical characteristics which may affect the pathogenesis and outcome of the HEV infection [1].

HEV is a major health concern in many developing countries from Asia and Africa, where major outbreaks occur due to the consumption of contaminated food or water, generally associated with HEV genotypes 1 and 2 [2]. These HEV variants infect primates, and are usually self-limited, but can cause fulminant hepatitis in pregnant women and potentially in patients with pre-existing liver diseases [2]. In developed and non-endemic countries, sporadic cases of HEV occur due to genotypes 3 and 4 of HEV. Many of these infections have been linked to ingestion of raw or undercooked meat of wild boar, pig, and deer, or to direct contact with these animals, which can be infected by these HEV variants, supporting the concept that transmission of these genotypes is zoonotic [3]. Interestingly, a recent report described a zoonotic transmission of HEV genotype 7 from the camel to human, in a solid organ transplant recipient, indicating that human infections with other genotypes could occur [4].

Little is known about HEV seroprevalence and its determinants in Iran. Considering the fact that Iran is among the countries in which HEV infection is endemic, population-based studies in this area of the world is justified. Studies on blood donors in Iran have demonstrated that the prevalence of HEV infection varies, ranging from 7.8% in Tehran, capital of Iran, and Tabriz, in East Azerbaijan Province, Eastern Iran, to 11.5% in Khuzestan province in the South of Iran [5–7].

In recent years, it has been found that individuals who are immunosuppressed, particularly solid organ transplant and also bone marrow transplant recipients, can develop chronic as well as acute HEV infection, mainly due to the virus persistence [1, 8, 9]. Recent studies regarding the HEV replication within the gut suggests incomplete clearance of the virus in this compartment, leading to endogenous reinfection when ribavirin therapy is stopped.

It has been shown, in several studies, that patients with HIV infection may acquire HEV infection more frequently than individuals without HIV [1, 10], On the other hand, several other studies have not shown differences in HEV prevalence between HIV-infected and non-infected individuals [1, 11]. The prevalence of HEV infection in HIV-infected patients ranged from nil up to 50% [1]. However, studies regarding the co-infection of HIV and HEV are limited and it is not clear whether HIV-infected patients are at greater risk of acquiring HEV infection due to shared modes of transmission or increased vulnerability due to immune suppression. Moreover, co-infected patients with a low CD4 T cell count are more likely susceptible to develop a chronic infection [12]. The present study aimed to determine the prevalence of HEV antigens and anti-HEV antibodies among HIV-infected patients in Fars Province, Southern Iran. Lack of information about the serostatus of HEV infection in HIV-infected subjects in most areas of Iran, including Fars Province in the south of the country, justified the current study.

## Materials and methods

### Patients and blood sampling

Subjects of this study were 251 HIV-confirmed patients (which had been confirmed by ELISA and Western blotting tests), admitted to Shiraz HIV/AIDS Research Center in 2017. The center is established for surveillance and monitoring of HIV-infected patients in Fars Province in the south of Iran. Respective data including patients’ demographic information were obtained for each patient. All the patients were clinically asymptomatic for HEV and received highly active antiretroviral therapy (HAART). Blood samples (5 mL) were collected from each patient. Sera were separated from the whole blood and stored at − 20 °C until use.

### Evaluation of hepatitis E antigens and anti-hepatitis E antibodies

The presence of HEV antibodies was assessed by a commercial ELISA kit (DIA.PRO, Diagnostic Bioprobes Sri, Milano, Italy, REF EVG.CE), based on the manufacturer’s instructions. For the detection of HEV antibodies, index value > 1.1 was regarded as positive. Index values less than 0.9 regarded as negative while those values between 0.9–1.1 were regarded as equivocal. Samples within the equivocal zone were re-tested. HEV antigen was detected by a commercial ELISA kit (MyBioSource, San Diego, California, United States, MBS412749) based on the manufacturer’s instructions. A cut off value, based on the mean absorbance value of three negative controls (supplied by the manufacture) was calculated. Samples giving an absorbance greater than, or equal to cut-off value were considered as positive.

### Statistical analysis

Statistical analysis was done using SPSS (version 18; SPSS Inc., Chicago, IL, USA). Chi-square or Fisher’s exact tests were used to find out the association between the seropositivity (either for HEV antigen or antibodies) and the socio-demographic or hematological features of the patients.

## Results

In this study, 251 confirmed cases of HIV positive patients were recruited. Out of 251 cases, 158 cases (62.9%) were male and 91 (36.2%) were female. Patients’ age varied from 14 to 83 (mean: 40 ± 9.7) years in which the majority (38.6%) were in 30 to 39 years of age group. The highest frequency (45.4%) of CD4+ T cell levels was seen in the 201–500 cell/μl group.

Serological evaluations of the subjects revealed that 26 (10.4%) of the HIV positive cases were positive for anti-HEV IgG antibodies and 6 (2.4%) were positive for HEV-antigens. Also, 2 (0.8%) of the patients were positive for both anti-HEV IgG antibodies and antigens. Among HIV positive male patients, 12% were positive for HEV antibodies and 3.2% for HEV antigen, whereas female HIV-infected patients had a seropositivity rate of 7.7% for HEV antibodies and 1.1% for HEV antigens. Statistical analysis, using the Chi-square test, revealed no significant association between sex and seropositivity to either HEV antigen (*P* = 0.420) or antibodies (*P* = 0.390). Considering the age of the participants, the highest HEV seropositivity for antibodies (38.6%) and for antigens (6.7%) was seen in the age group of 30–39 and 50–59 years, respectively. No significant association was seen between age groups and seropositivity for anti-HEV antibodies or antigen (*P* = 0.622 and 0.945, respectively). Among different CD4+ T cell level groups, the highest prevalence of HEV antibodies (13.3%) and antigen (2.7%) were seen among the CD4+ T cell levels of 101–200 and > 500, respectively. The association between CD4+ T cell level and seropositivity to HEV was not significant (*P* = 0.687 and 0.911, respectively). Table 1 shows the socio-demographic and immunological features of HIV patients and relative seropositivity to HEV antigen or antibody in this study.

**Table 1:** Socio-demographic and immunological features of HIV patients and relative seropositivity to HEV antigen or antibody in Fars Province, southern Iran

Characteristics	Frequency (No.)	Percent (%)	Positive for anti-HEV antibodies		Positive for HEV antigens	
			No.	%	No.	%
^a^Gender						
Male	158	62.9	19	12	5	3.2
Female	91	36.3	7	7.7	1	1.1
Age groups						
< 30	29	11.6	2	6.9	1	3.4
30–39	97	38.6	11	11.3	3	3.1
40–49	93	37.1	10	10.7	1	1.1
50–59	15	6	1	6.7	1	6.7
> 60	17	6.8	2	11.8	0	0
CD4+ count (cell/μl)						
0–100	19	7.6	2	10.5	0	0
101–200	45	17.9	6	13.3	1	2.2
201–500	114	45.4	9	7.9	3	2.6
> 500	73	29.1	9	12.3	2	2.7
Total	251	100	26	10.4	6	2.4

^a^ 2 patients are missing in the sex description

## Discussion

Susceptibility of HIV positive individuals to HEV infection is a controversial issue. Several studies have shown relatively high seroprevalence of HEV in HIV-infected patients, [13] while some studies have not shown such association [14]. The frequency of HEV infection in HIV-infected patients is reported to be more than 40% in some regions of Africa and Asia, 10 to 20% in EU countries and less than 10% in the US and Oceania [1].

A high prevalence of anti-HEV antibodies in HIV-infected subjects has been reported in several studies. A study on 613 HIV-infected patients in Spain revealed seropositivity of 26% for anti-HEV antibodies [15]. Boon et al., reported that 47% of HIV-infected patients in Uganda are seropositive for anti-HEV antibodies [10]. Shrestha et al., reported a high prevalence (39.4%) of anti-HEV antibodies in Nepalian HIV-positive subjects [16]. Moreover, the seroprevalence of HEV in Nigerian HIV-infected individuals was reported to be 20% [17]. In Italy, Rapicetta et al., reported that 19.4% of HIV-infected subjects are seropositive for HEV antibodies [18]. Also, Madden et al. reported that 23.3% of HIV-infected patient in South Africa have IgG antibodies against HEV [19].

Iran is located in the south of Asia and is classified among the endemic regions for HEV infection, where outbreak of HEV infection has been recorded [20]. The current study was conducted in Fars province, southern Iran, in which a seroprevalence of 10.4% was found for anti-HEV antibodies. Our findings are in consistent with the findings of a previous study in 2013 by Joulaei et al., on 153 HIV-infected patients in Shiraz, capital of Fars province, in which the seroprevalence of HEV antibodies in HIV-infected individuals was reported to be 16.4% [21]. Our results were also similar to previous studies on HIV patients by Ramezani in 2012 in Tehran, capital of Iran, in which HEV antibodies were detected in 10% (10 out of 100 cases) of HIV-infected patients [11].

A study conducted by Asaei et al., in 2015 on 1030 sera samples from the general population in Fars province in the south of Iran, revealed a seroprevalence rate of 13.4% for anti-HEV antibodies [22]. In a similar study, conducted by Taremi et al., on 1824 cases of the general population in Nahavand, West of Iran, a seroprevalence rate of 9.3% has been reported for anti-HEV antibodies [23]. Moreover, studies on blood donors in different areas of Iran including Tehran, [24] Azerbaijan, [25] and Kerman provinces, [26] showed relatively low seroprevalence (7.8, 7.8, and 7.7%, respectively) for HEV infection. By comparing the findings of these studies with our findings, it can be suggested that HIV positive patients are not at greater risk of HEV infection than the general population. However, the rate of antigen seropositivity in the general population has not been ascertained in the aforementioned studies and further studies are needed to find out the rate of antigen seropositivity in the general population in comparison with HIV-infected individuals.

It has been shown that HEV antigen is detectable in the serum at almost the same time as HEV RNA in feces, but persisted for four weeks less than HEV RNA [27]. In a study by Mishra et al. it was documented that HEV antigen detection can be used as a valuable marker of active viremia and a cheaper surrogate to HEV real-time PCR, particularly in the window period, in pregnant women and immunocompromised patients [28]. In our study, ELISA was used to detect HEV antigen in HIV-infected individuals, where 2.4% of the cases were seropositive. This indicates that a relatively low percent of HIV-infected individuals in our study have acute or chronic HEV infection. In our study, the HIV-positive patients have been subjected to HAART therapy. There might be a possibility that active HAART therapy represses the expression of HEV antigens and thus affects the percentage of HIV/HEV co-infection. Evaluation of HEV antigen-positive cases by molecular methods provides appropriate data about the genotypes of HEV in the seropositive subjects. The viral RNA was not measured in our study and this can be considered as a drawback of our study.

Correlation between sex and HEV infection is a debated issue in which some studies showing no correlation between the two sexes [29, 30]. A study by Faber et al. on 4422 adults’ population in Germany showed no significant difference in seroprevalence of HEV between sexes [30]. Consistent with this study, in our study, the association between gender and HEV seropositivity was not significant. Nevertheless, a few studies are showing a significant correlation between HEV infection and gender [19, 20].

## Conclusion

The results of the current study showed a relatively low prevalence of anti-HEV antibodies in HIV-infected patients, indicating that HIV positive patients may not be at greater risk of HEV infection than the general population. Moreover, HEV-antigen was detected in a few cases of HIV-infected individuals which may indicate acute or chronic HEV infection in these patients. Further studies are needed to find out the rate of antigen seropositivity in HIV-infected individuals in comparison with the general healthy population.

## Data Availability

All data generated or analyzed during this study are included in this manuscript.

## Abbreviations

HBV: Hepatitis B virus
HCV: Hepatitis C virus
HEV: Hepatitis E virus
HIV: Human immunodeficiency virus
HAART: Highly active antiretroviral therapy
